# Protein secretion and surface display in Gram-positive bacteria

**DOI:** 10.1098/rstb.2011.0210

**Published:** 2012-04-19

**Authors:** Olaf Schneewind, Dominique M. Missiakas

**Affiliations:** Department of Microbiology, University of Chicago, 920 East 58th Street, Chicago, IL 60637, USA

**Keywords:** type VII secretion, WXG protein, sortase, sorting signal, surface-layer homology domain, surface layer

## Abstract

The cell wall peptidoglycan of Gram-positive bacteria functions as a surface organelle for the transport and assembly of proteins that interact with the environment, in particular, the tissues of an infected host. Signal peptide-bearing precursor proteins are secreted across the plasma membrane of Gram-positive bacteria. Some precursors carry C-terminal sorting signals with unique sequence motifs that are cleaved by sortase enzymes and linked to the cell wall peptidoglycan of vegetative forms or spores. The sorting signals of pilin precursors are cleaved by pilus-specific sortases, which generate covalent bonds between proteins leading to the assembly of fimbrial structures. Other precursors harbour surface (S)-layer homology domains (SLH), which fold into a three-pronged spindle structure and bind secondary cell wall polysaccharides, thereby associating with the surface of specific Gram-positive microbes. Type VII secretion is a non-canonical secretion pathway for WXG100 family proteins in mycobacteria. Gram-positive bacteria also secrete WXG100 proteins and carry unique genes that either contribute to discrete steps in secretion or represent distinctive substrates for protein transport reactions.

## 1. Introduction

Hans Christian Gram used light microscopy to detect microbes that were stained with crystal violet/iodine [1]. Microbes that cannot retain this dye following treatment with ethanol were counterstained with safranin (or fuchsin), thereby distinguishing Gram-positive from Gram-negative bacteria. The differential staining property is based on the peptidoglycan layer, which is considerably thicker in Gram-positive microbes [2]. Another difference is that Gram-positive bacteria elaborate a single membrane, whereas Gram-negative microbes harbour a plasma membrane and an additional outer membrane with lipopolysaccharides [3,4]. The secretion of signal peptide-bearing precursor proteins in Gram-positive microbes leads by default to their release into extracellular medium [5]. In contrast, signal peptide-mediated translocation in Gram-negative bacteria leads to protein localization within the periplasm, a compartment between their inner and outer membranes that encompasses a thin peptidoglycan layer [6].

In the past few years, molecular biology approaches have been applied to many Gram-positive bacteria and the ensuing research evolved into some of the most exciting fields of microbiology and microbial pathogenesis, including *Actinomyces* spp., *Bacillus anthracis*, *Bacillus cereus*, *Bacillus subtilis*, *Clostridium perfringens*, *Clostridium difficile*, *Corynebacterium diphtheriae*, *Enterococcus faecalis*, *Enterococcus faecium*, *Listeria monocytogenes*, *Streptococcus agalactiae*, *Streptococcus gordonii*, *Streptococcus pyogenes* and *Streptomyces* spp. This review summarizes briefly what is known about the secretion or assembly of proteins in the envelope of Gram-positive microbes. Our goal was to provide a comparative synopsis of parallel fields that may benefit from each other and to define key research frontiers. Because our discussion had to be brief, we apologize for not being able to provide a more exhaustive analysis and for not discussing many important advances in each field.

## 2. Envelope structures in Gram-positive bacteria

Peptidoglycan is synthesized from nucleotide precursors [7], the modified amino sugar *N*-acetylmuramic acid (MurNAc) [8] as well as d- or l-amino acids (l-Ala-d-iGlu-*m*-Dpm-d-Ala-d-Ala, here abbreviated AGDA_2_) [9] in the bacterial cytoplasm to generate Park's nucleotide (UDP-MurNAc-AGDA_2_) [10]. Park's nucleotide is linked to the lipid carrier undecaprenyl-pyrophosphate to generate lipid I [C_55_-PP-MurNAc-AGDA_2_] [11]. Modification of lipid I with UDP-GlcNAc produces lipid II [C_55_-PP-MurNAc(AGDA_2_)-GlcNAc] [12], which is polymerized by transglycosylases and transpeptidases (penicillin-binding proteins) to generate peptidoglycan strands [MurNAc(AGDA_2_)-GlcNAc-MurNAc(AGDA_2_)-GlcNAc] that are crosslinked with other strands [13,14].

**Figure 1.**
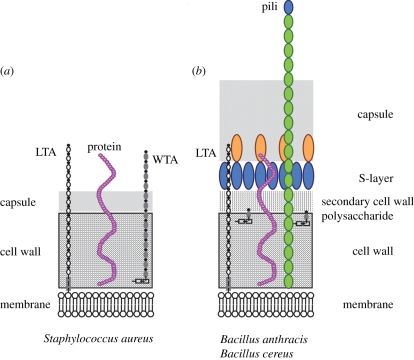
Envelope structures of Gram-positive bacteria. (*a*) *Staphylococcus aureus* elaborates a plasma membrane and thick peptidoglycan layer that encompasses polyribitol-phosphate wall teichoic acids (WTA), proteins and capsular polysaccharides. Lipoteichoic acids (LTA) are poly-glycerolphosphates tethered to a diglucosyl-diacylglycerate membrane anchor. (*b*) *Bacillus cereus* as well as *Bacillus anthracis* elaborate a plasma membrane and thick peptidoglycan layer. Secondary cell wall polysaccharides (SCWPs) are linked to peptidoglycan and serve as a ligand for the SLH domains of S-layer proteins. In addition to cell wall anchored proteins, the peptidoglycan of bacilli also functions as an anchoring point for capsule and pili.

The peptidoglycan layer protects Gram-positive bacteria from osmotic lysis and serves as a barrier against environmental hydrolases or membrane toxic compounds [2]. Peptidoglycan also functions as a scaffold for the immobilization of capsular polysaccharides [15], cell wall teichoic acids (WTAs) [16] and proteins that are either bound to specific envelope structures [17,18], covalently linked to peptidoglycan [19], assembled into pili [20] or distributed into S-layer structures [21,22]. Proteins in any one of the aforementioned locations fulfil unique physiological roles that aid bacteria in their interaction with the environment, most notably the tissues and immune cells of an infected host [23] (figure 1).

## 3. Protein translocation across the plasma membrane

The genetic requirements for the secretion of signal peptide-bearing precursors have not been examined in Gram-positive bacteria. What has been learned from *Escherichia coli* suppressor analyses on the involvement of *sec* genes is thought to apply also to bacilli, staphylococci or any other Gram-positive microbe [24,25]. Further, *in vitro* translocation experiments with inverted membrane vesicles of *E. coli* analysed the biochemical attributes of the Sec pathway [26]. In brief, these lines of investigation identified SecYEG as the translocon channel for precursor movement across the plasma membrane [27], which also involves the cytoplasmic ATPase SecA for pushing precursor proteins into the channel [28] and the SecDF/YajC complex for releasing them from the translocon [29]. Signal peptidase acts on the translocated precursor to release the mature polypeptide into the periplasmic space [30]. The signal recognition particle (SRP) of *E. coli* is a ribonucleoprotein complex comprising Ffh and 4.5S RNA, which interacts with the nascent precursors of membrane proteins to regulate translation and deliver the ribosome to the SRP receptor (FtsY) and eventually the SecYEG translocon for co-translational secretion of membrane proteins [31,32]. Several chaperones, including SecB [33], heat shock proteins (DnaK/DnaJ/GrpE) [34] as well as trigger factor, a peptidyl-prolyl isomerase [35], contribute to secretion by maintaining specific substrate proteins in a secretion competent state [4,36].

Most Gram-positive bacteria harbour *secA*, *secD*, *secE*, *secF*, *secG*, *secY*, *ffh*, *ftsY* and *yajC* genes and are thought to catalyse protein secretion by pathways similar to those described for *E. coli* [37]. A notable difference is that Gram-positives lack the *secB* gene and require *prsA*, a lipoprotein peptidyl-prolyl isomerase for secretion of some polypeptides into the extracellular media [37,38]. Some Gram-positive bacteria express accessory secretion genes designated *secA2* or *secY2*. In *S. gordonii*, the *secA2* and *secY2* gene products are essential for the secretion of the large glycoprotein GspB, which, owing to its heavy glycosylation, cannot be transported via the SecA pathway [39,40]. In addition to its 90 residue N-terminal signal peptide, the first 20 amino acids of mature GspB are required for SecA2/SecY2-mediated translocation by a process that involves also the accessory secretion proteins Asp1–5 [41,42]. Several Gram-positive microbes also use accessory secretion genes, *secA2* and *secY2* (either alone or together), for the selective transport of specific substrates [43]. Unlike *E. coli* [44], the SRP pathway of *Streptococcus mutans* is dispensable for the growth of this bacterium [45,46]. Nevertheless, *ffh, ftsY* or *scRNA* mutants display defects in acid tolerance and ATPase activity [45,46]. Some Gram-positive microbes harbour two genes encoding YidC homologues (YidC1 and YidC2) where one of the YidC isoforms appears to specialize in a cotranslational secretion pathway that operates independently of SRP [47].

## 4. Protein traffic across the cell wall and its associated structures

The peptidoglycan layer of Gram-positive bacteria is much thicker than that of Gram-negative microbes; in some cases, it can be 50–100 nm in diameter [48]. Cell wall envelopes can be isolated by first physically breaking cells with glass or aluminium beads and then purifying murein sacculi, which are impenetrable to proteins [49]. One wonders whether some Gram-positive bacteria have evolved channels for the release of precursors that have been translocated across the plasma membrane into the extracellular milieu. A simple argument in favour of protein transport channels across peptidoglycan is the finding that boiling staphylococci in hot SDS does not release membrane or lipoproteins from the murein sacculus [50]. However, puncturing murein with specific hydrolases does provide for the detergent extraction of such lipoproteins. The peptidoglycan layer functions also as a surface organelle that enables assembly of capsules [51], secondary cell wall polysaccharides (SCWPs) and S-layers [22,52,53], WTAs [16] and of many different sortase-anchored proteins [23,54]. Whether peptidoglycan associated polymers of proteins, polysaccharides or teichoic acids provide a barrier to protein translocation has not been studied.

Using electron microscopy and immunogold labelling techniques in *S. pyogenes*, Rosch & Caparon [55,56] reported the accumulation of immune-reactive signals at membrane sites (microdomains) that include SecA and HtrA (DepP). Both of these proteins are involved in precursor protein secretion in *E. coli* and fulfil similar functions in streptococci [57]. The authors proposed the existence of a macromolecular structure designated the ExPortal, which could be responsible for the secretion of precursor proteins in Gram-positive microbes with thick peptidoglycan layers [56]. In agreement with this hypothesis, similar accumulations of immune-reactive membrane signals have been observed in *E. faecalis* [58]. The molecular compositon, structural features or genes responsible for the formation of the proposed ExPortal have not yet been revealed. The ExPortal hypothesis has been challenged. Others reported that SecA of *S. pyogenes* is not localized to microdomains but distributed throughout the streptococcal membranes [59].

## 5. Protein secretion into the cross wall of Gram-positive cocci

Bioinformatic analyses identified two types of signal peptides in genes for precursor proteins of *Staphylococcus aureus*, *Streptococcus pneumoniae* and *S. pyogenes* [60–62]. Lindahl and co-workers [59] discovered that M protein, a cell wall anchored surface protein of *S. pyogenes* that is secreted via a signal peptide harbouring the YSIRK/GS sequence motif, is deposited near the cell division site, whereas protein F, whose precursor harbours a conventional signal peptide, is deposited near the cell poles. The unique distribution of surface proteins is imposed by the two types of signal peptides, as its switching redirects mutant M protein and protein F precursors to the other location [59]. A similar phenomenon has been reported for *S. aureus* where precursors with YSIRK/GS signal peptides are secreted into the cross wall, the peptidoglycan synthesis compartment that is formed at midcell following FtsZ-mediated cytokinesis [63]. Surface proteins that are targeted into the cross wall because of their YSIRK/GS signal peptides are eventually distributed over the entire bacterial surface, whereas those that are immobilized following secretion via canonical signal peptides reside only at the cell poles [63,64].

Recent work identified genes for membrane proteins with abortive-infectivity domains, designated *spdABC* (surface protein display), as being required for the trafficking of YSIRK/GS proteins into the cross wall compartment [65]. Mutants that lack any one of the three *spdABC* genes display increased thickness of the cross wall compartment and delayed cell separation during staphylococcal cell division [65]. LytN is a murein hydrolase that is secreted into the cross wall of *S. aureus* via its YSIRK/GS signal peptide [66]. Staphylococcal *lytN* mutants display cell separation and cross wall structural defects, suggesting that LytN plays a key role in completing cross wall formation and cell separation [66]. Two other secreted murein hydrolases, Sle1 and Atl, also contribute to cross wall separation [67,68]. These enzymes act on the outside the cell wall envelope in the immediate vicinity of the cell division site and split the cross wall from the outside [69,70]. Although these experiments point to various secreted products that are essential in cell wall synthesis and cross wall physiology, the fundamental question of how precursors with YSIRK/GS signal peptides are secreted into the cross wall compartment has not yet been addressed.

## 6. Sortase A-anchoring of proteins to the cell wall envelope

**Figure 2.**
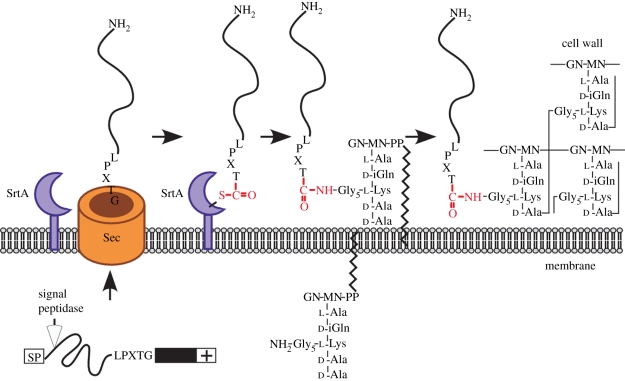
The role of sortase A in anchoring proteins to the cell wall envelope of Gram-positive bacteria. Surface proteins are synthesized as precursors with an N-terminal signal peptide and a C-terminal LPXTG motif sorting signal, including a hydrophobic domain (black box) and positively charged tail (+). Following initiation into the secretory pathway, the signal peptide is cleaved, while sortase A scans translocated precursors for LPXTG motif sequences. Following cleavage of the precursor by sortase A between the threonine (T) and the glycine (G) of the LPXTG motif, an acyl-enzyme intermediate is formed between the active site cysteine (C) of sortase A and the carboxyl-group of threonine at the C-terminal end of the surface protein. The amino group of cell wall crossbridges within lipid II, pentaglycine (Gly_5_) in *S. aureus*, performs a nucleophilic attack at the thioester bond between sortase A and its cleaved substrate, thereby forming an amide (isopeptide) bond between the C-terminal threonine and lipid II. Transglycosylation and transpeptidation reactions promote the incorporation of surface proteins into the cell wall envelope of Gram-positive bacteria (see text for details). MN and GN denote *N*-acetylmuramic acid and *N*-acetylglucosamine, respectively.

All Gram-positive bacteria express sortase A, a membrane anchored transpeptidase that cleaves the C-terminal LPXTG sorting signals of precursor proteins destined for cell wall anchoring [71,72]. Sortase A scans secreted polypeptides for its recognition sequence with a unique fold of parallel and anti-parallel β-sheets [73] (figure 2). A scissile peptide bond between the threonyl and the glycyl of LPXTG motif sorting signals is accommodated in direct proximity of the active site cysteine of sortase A [74]. Following nucleophilic attack from the active site, sortase forms a thioester linked intermediate with the carboxyl group of threonine at the C-terminal end of surface proteins [72]. The sortase acyl intermediate is resolved by the nucleophilic attack of the free amino group within lipid II, thereby releasing surface protein linked to the crossbridge and restoring the enzyme active site as reaction products [75,76]. The crossbridge of lipid II precursors varies in structure between bacterial species [77]. In bacilli and listeria, the crossbridge amino group is derived from the side chain of *m*-diaminopimelic acid (*m*-Dpm). In contrast, the crossbridge of staphylococci comprises five glycyl residues that are tethered to the *ɛ*-amino group of lysine within lipid II [C_55_-(PO_3_)_2_-MurNac(l-Ala-d-iGln-Lys(NH_2_-Gly_5_)-d-Ala-d-Ala)-GlcNAc] [78,79]. Sortase A enzymes of different bacterial species have evolved to recognize their corresponding crossbridge structures of lipid II [80,81]. The sortase A reaction products, surface protein linked to lipid II, are then incorporated into the cell wall envelope via the transglycosylation and transpeptidation reactions of cell wall synthesis [82–84].

*Staphylococcus aureus* sortase A mutants (*srtA*) are unable to anchor any one of 19 surface proteins with LPXTG sorting signals to the cell wall envelope [85]. These sortase A mutants display a large defect in virulence as the variants cannot cause lethal sepsis or form abscesses in mouse models for staphylococcal disease [86–88]. Sortase-anchored products play an important role in the pathogenesis of disease caused by many different Gram-positive pathogens [54]. There are, however, also some exceptions. For example, *B. anthracis* sortase A mutants do not display significant virulence defects [89], whereas proteins in the S-layer, for example, the S-layer-associated protein BslA, make significant contributions to the pathogenesis of anthrax [90,91].

The distribution of sortase A in the envelope of *S. pyogenes* was examined with fluorescence microscopy at various stages of cell growth [92]. During cell division, most sortase A molecules can be found in the cross wall compartment but also at polar sites of surface protein anchoring [92]. This accumulation decays once cell division is complete. Surprisingly, by applying electron microscopy and immunogold labelling techniques, sortase A could be observed at a single site within the plasma membrane of *E. faecalis* [58]. As this location coincided with the location of pilin-specific sortase, sortase substrates and SecA, sortase A has been proposed to associate with the aforementioned ExPortal structure [58]. The discrepancy of the data on sortase localization for *S. pyogenes* and *E. faecalis* can currently not be reconciled.

## 7. Sortase B-anchoring of haem scavenging factors

**Figure 3.**
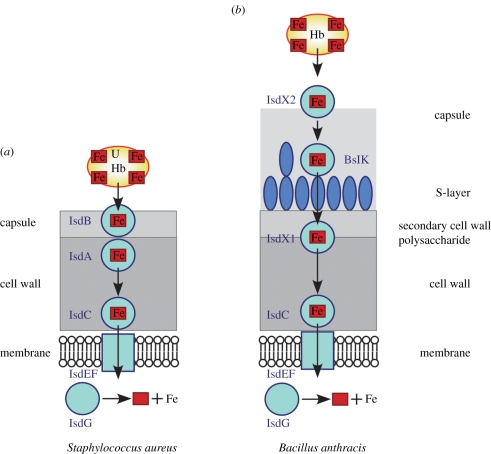
Haem-iron scavenging in Gram-positive bacteria. (*a*) The IsdB haemophore of *S. aureus* removes haem (red box Fe) from haemoglobin (Hb) and transfers the liberated compound to IsdA; both proteins are linked to the cell wall by sortase A. IsdA, in turn, transfers haem to IsdC, a sortase B anchored product that is located in the vicinity of the plasma membrane. Transfer of haem from IsdC to the membrane transporter IsdEF enables staphylococcal import of haem and cleavage by the mono-oxygenase IsdG, which liberates iron (Fe) from haem for staphylococcal growth. (*b*) *Bacillus anthracis* secretes IsdX1 and IsdX2, which function as haemophores and haem transfer units. IsdX2 enables transfer of haem to BaslK, an S-layer protein, and then to IsdC. *Bacillus anthracis* sortase B promotes the anchoring of IsdC to the cell wall envelope. Following haem import across the plasma membrane, the tetrapyrrole is cleaved by IsdG and iron (Fe) is liberated.

The gene for sortase B (*srtB*) is located within the iron-regulated surface determinant (*isd*) gene cluster of bacilli, clostridia, listeria and staphylococci [93]. In *S. aureus*, the cluster (*isdA*–*isdB*- *isdCDEF*-*srtB*-*isdG*) encodes for two sortase A anchored products, IsdA and IsdB, as well as three proteins in the plasma membrane (IsdD and the ABC transporter IsdEF) that transport haem across the plasma membrane [93] (figure 3). Haem-binding NEAr-iron Transporter (NEAT) domains are a common feature of IsdA, IsdB, IsdC and IsdD [93]. IsdG is a cytosolic protein with a unique structure that cleaves the tetrapyrrole ring of haem to release iron for bacterial growth [94,95]. Sortase B cleaves the NPQTN sorting signal of a single substrate protein, eIsdC [85]. SrtB assumes a similar fold and catalytic reaction mechanism as SrtA [96]; however, the enzyme appears to accept the crossbridge amino groups of assembled cell wall as a nucleophile to anchor IsdC in the immediate vicinity of the membrane [97]. SrtB activity and the differential distribution of IsdC compared with the SrtA-anchored products (IsdA and IsdB) enable passage of haem-iron across the bacterial cell wall envelope [98]. IsdB as well as IsdH, another SrtA-anchored protein whose gene is located elsewhere on the bacterial chromosome, remove haem from the host proteins haemoglobin and haptoglobin and transfer the polypeptide to IsdC [93,99–101]. IsdC in turn transfers haem to the membrane protein IsdD for subsequent import of the nutrient into the bacterial cytoplasm [102–104].

Several other Gram-positive microbes express sortase B homologues and have evolved *isd* gene clusters to accommodate their unique envelope structures [98]. For example, *B. anthracis* sortase B anchors IsdC to the bacterial peptidoglycan by cleaving its NPKTG sorting signal [105]. Bacilli secrete two haemophores, IsdX1 and IsdX2, into the envelope as well as the extracellular milieu [106]. BasK is a NEAT domain protein in the S-layer of *B. anthracis* [107]. Haem scavenging of bacilli requires the haemophore activities of IsdX1 and IsdX2 [106], followed by haem transfer to BasK and then to IsdC [107]. Following haem import across the plasma membrane by the ABC transporter IsdEF, the tetrapyrrole ring structure of haem is cleaved by IsdG [108,109].

## 8. Sortase C-anchoring of proteins to the envelope of spores

**Figure 4.**
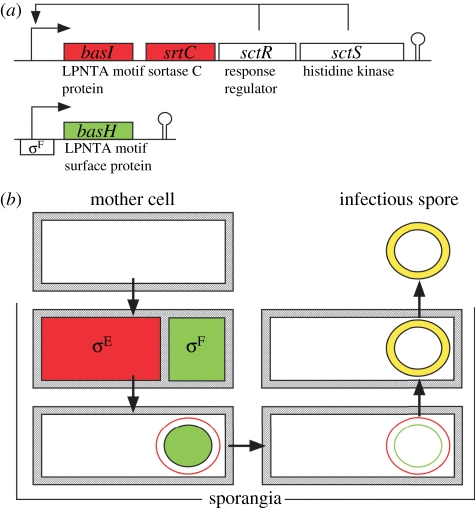
Role of sortase C in anchoring *B. anthracis* proteins to the envelope of sporangia or forespores. (*a*) During sporulation, *B. anthracis* activates the expression of *basI* and sortase C (*srtC*) in the mother cell compartment via the two component regulatory system *sctRS*. The *basH* gene is expressed via σ^F^-RNA polymerase in the developing forespore. (*b*) Sortase C cleaves the LPNTA motif sorting signals of BasI and BasH, anchoring the two polypeptides to the cell wall peptidoglycan of mother cells or the developing forespore, respectively. Sortase C ensures the formation of infectious spores in carcass tissues, i.e. under conditions where oxygen has become limiting.

The genes for sortase C and its homologues are found only in spore-forming microbes, including *B. anthracis* and *Streptomyces coelicolor* [110]. In *B. anthracis*, the sortase C gene (*srtC*) is part of the *basI*-*srtC*-*sctR*-*sctS* operon, which is expressed in a manner requiring the two component regulator SctRS during the onset of sporulation [111] (figure 4). Expression of *srtC* is initiated precisely 2 h prior to the establishment of the asymmetric septum that initiates *B. anthracis* spore formation in the carcasses of animals that have succumbed to anthrax disease [111]. SrtC products are first detected in the plasma membrane of sporulating bacilli and, following asymmetric cell division, are subsequently inherited by the mother cell and its endspore [111]. The *basI* gene product is anchored by SrtC to the peptidoglycan crossbridges of the mother cell envelope [112]. A second substrate gene, *basH*, is expressed via σ^F^ RNA polymerase only in the forespore compartment [111]. BasH precursor is secreted across the plasma membrane into a peptidoglycan compartment bounded by both endospore and mother cell membranes [111]. SrtC cleaves the LPNTA motif of BasI and BasH between the threonyl and the alanyl residues of their LPNTA sorting signals [111,112]. Its acyl-enzyme intermediate accepts the *m*-Dpm of crossbridges as a nucleophile for the anchoring of mature proteins to either the mother cell (BasI) or the endospore (BasH) peptidoglycan [112]. Sortase C is required for the formation of infectious *B. anthracis* spores [111].

*Streptomyces coelicolor*, a high GC content Gram-positive, filamentous soil bacterium, forms mycelia that submerge in liquid or semiliquid environments [113]. The mycelia differentiate to generate aerial hyphae that septate and then develop spore chains for their eventual dissemination in air [114]. Hyphal surfaces are highly hydrophobic, a property that promotes outgrowth into the air and dispersion of spores. Chaplins, a family of surface proteins, are required for aerial hyphae formation, and their anchoring to the cell wall envelope is catalysed by a homologue of sortase C [113,114]. Presumably, chaplins function to lower the aqueous surface tension for the emergence of aerial hyphae [113,114]. Of note, *B. anthracis srtC* anchored products fulfil a similar role, as sporulating bacilli must satisfy their requirements for oxygen to complete a developmental programme in carcasses where the circulation of oxygenated blood has stopped [111]. Although this has not been shown directly, it is presumed that SrtC anchored products enable bacilli to access air–tissue interfaces.

## 9. Pilus assembly in Gram-positive bacteria

**Figure 5.**
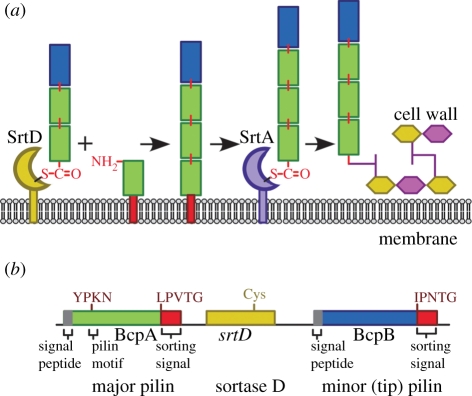
Pilus-specific sortase catalyses the polymerization of pili in *B. cereus* and other Gram-positive bacteria. (*a*) Pilus-specific sortase (SrtD) cleaves the LPVTG and IPNTG sorting signals of the major (BcpA) and minor (BcpB) pilins, respectively. The acyl-enzyme intermediate is resolved by the nucleophilic attack of the *ɛ*-amino group of lysine within the YPKN pilin motif. The sorting signal of BcpA, but not of BcpB, can be cleaved by sortase A (SrtA), which promotes cell wall anchoring and effectively terminates pilus assembly. (*b*) Pilus genes are organized into clusters encompassing the major and minor pilin genes with signal peptides, sorting signals and the YPKN pilin motif (major pilin), as well as the pilus-specific sortase. The structural gene for sortase A (*srtA*) is located elsewhere on the chromosome.

Several different Gram-positive bacteria elaborate pili via a sortase-assembly mechanism, including *Actinomyces* spp*.* [115], *B. cereus* [116], *C. diphtheriae* [20], *Enterococcus* spp*.* [117], *Lactobacillus rhamnosus* [118], *S. agalactiae* [119], *Streptococcus gallolyticus* [120], *S. pneumoniae* [121] and *S. pyogenes* [122] (reviewed in Kang *et al*. [123] and Hendrickx *et al*. [124]). Gram-positive pili are composed of pilin proteins, whose precursors harbour N-terminal signal peptides and C-terminal sorting signals [20]. The shaft of these 0.1–2 µm pilus filaments is composed of subunits of the major pilin protein [20]. The tip of each filament is decorated with a minor pilin, which promotes attachment of bacteria to host tissues [20,125]. Some, but not all, pili of Gram-positive bacteria harbour yet another pilin subunit, which resides at the base of filaments, providing a link with the cell wall envelope [126]. In microbes whose pili are elaborated from one major and one minor pilin, the filament is tethered to the cell wall envelope through a link with the final major pilin subunit at the base of the structure [127]. For example, pilus assembly in *B. cereus* involves two pilins, the major (BcpA) and minor (BcpB) subunits, as well as two sortase enzymes [116] (figure 5). The pilin-specific sortase (SrtD) recognizes as a nucleophile the *ɛ*-amino group of a conserved lysine residue (K) within the YPKN pilin motif, which is present only in BcpA but not in BcpB [128]. SrtD cleaves the C-terminal sorting signals of both BcpA and BcpB to generate acyl-enzyme intermediates [128]. These enzyme intermediates can only be resolved through the nucleophilic attack of the YPKN pilin motif, thereby generating amide (isopeptide) bonds between the C-terminal threonine of the LPXTG sorting signal and the lysine side chain of the YPKN motif in another pilin subunit (intermolecular amide bonds) [128,129]. The second enzyme essential for pilus assembly is sortase A, which recognizes the lipid II amino group as a nucleophile for its acyl-enzyme intermediates [127]. A key feature of *B. cereus* pilus assembly is the BcpA sorting signal, which has evolved as a substrate for both SrtD and SrtA [128]. Sortase A cleavage of the BcpA sorting signal within a growing pilus filament leads to cell wall anchoring of the pilus, effectively ending the polymerization of pilins. In other words, the substrate properties of BcpA for two sortases are determinants of pilus polymerization and length [130]. The sorting signal of BcpB cannot be cleaved by sortase A [129]. The SrtD acyl intermediate with BcpB requires the BcpA nucleophile for resolution, a substrate property that ensures BcpB deposition at the tip of pili [129].

*Corynebacterium diphtheriae* assemble pili from three subunits [131]. The sorting signals of the major pilin (SpaA) and the tip adhesin (SpaC) are cleaved by pilin-specific sortase [20]. Here, too, pilus assembly occurs through the nucleophilic attack of the *ɛ*-amino group of lysine within the YPKN pilin motif of the major pilin (SpaA). SpaB is cleaved by the house-keeping sortase of corynebacteria and anchored to the cell wall envelope [126,132]. A unique feature of SpaB is its substrate property of presenting the *ɛ*-amino group of a lysine residue as a nucleophile to pilin-specific sortases [126]. This mechanism ensures that transfer of polymerized pili to SpaB leads to the cell wall anchoring of the polymerized structure [126].

X-ray crystallography of recombinant (non-polymerized) pilin subunits provided key insights into the assembly of pili and surface proteins of Gram-positive bacteria [133]. Gbs52, the minor pilin from *S. agalactiae*, folds into two adjacent immunoglobulin (Ig)-like domains with seven anti-parallel β-strands [134]. One of the two Ig-like domains harbours an intramolecular isopeptide bond [134]. Similar intramolecular bonds were identified in the major pilin subunit of *S. pyogenes* (Spy0128) [133]. These bonds are formed autocatalyically from the side chains of lysine (Lys) and asparagine (Asp) residues via a mechanism requiring close proximity of the carboxylate of glutamic acid (Glu) or aspartic acid (Asp) [123,133,135]. Intramolecular isopeptide bonds are a stabilizing feature of Cna-B type domains, i.e. Ig-like domains that were first described in the Cna surface protein of *S. aureus* [136]. Cna-B type domains as well as other folds with intramolecular isopeptide bonds are found in many surface proteins of Gram-positive bacteria [133]. They appear to function as stabilizers of secreted proteins, which, in many Gram-positive bacteria, cannot acquire disulphide bonds, as occurs via the DsbAB pathway for proteins secreted into the periplasm of Gram-negative bacteria [137]. X-ray crystallography also provided insights into the catalysis of pilus assembly by revealing the structure of pilin-specific sortases [138]. The overall structure of pilin-specific sortases is similar to that of sortase A and sortase B [74,138,139]. A distinguishing feature of the pilin-specific sortases of *S. pneumoniae* and *S. agalactiae* is a flexible lid that covers the active site of the enzyme [138,140]. The contributions of the lid towards sortase recognition of the side chain of lysine within the YPKN motif of major pilins are not yet appreciated. X-ray crystallography of Spy0129, the pilus-specific sortase of M1 strain *S. pyogenes* SF370 (SrtC1) revealed a structure more similar to that of sortase B and without a lid over the active site [141,142]. Current work on pilus formation in Gram-positive bacteria focuses on *in vitro* assembly assays and a complete structural appreciation of how sortases recognize pilin subunits and assemble the pilus fibre [143–145].

## 10. S-layer assembly in bacilli and clostridia

Surface layers (S-layers) are para-crystalline sheets of proteins that assemble on the surface of bacteria [21,146]. Bacteria elaborate S-layers by abundantly secreting one or two proteins that self assemble into a two-dimensional lattice on the microbial surface [147]. Other proteins associate with the main S-layer proteins and fulfil variable functions in that they act either as a scaffold or enzyme in the bacterial envelope [148], promote nutrient diffusion or transport [107], or contribute to virulence by enabling microbial adhesion to infected host tissues [91]. S-layer proteins and S-layer-associated proteins of many bacteria share three tandem approximately 55 amino acid repeats of the surface-layer homology (SLH) domain [149–151]. Secreted proteins harbouring three tandem SLH domains are tethered to the bacterial envelope by non-covalent interactions between the SLH domains and a secondary cell wall carbohydrate [22]. SLH domains are remarkable for being both necessary and sufficient for the incorporation of chimeric proteins into S-layers [152].

**Figure 6.**
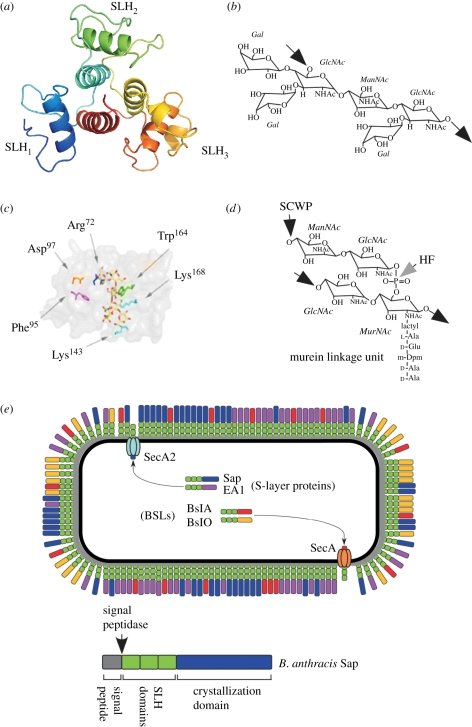
S-layer assembly occurs through the association of secreted proteins with SLH domains and the SCWP. (*a*) Three pronged spindle structure of the SLH domains of S-layer and S-layer-associated proteins in *B. anthracis*. (*b*) Structure of the SCWP of *B. anthracis*. (*c*) Conserved residues of the SLH domains of bacilli and the location within the inter-prong grooves of SLH domains. The SCWP was modelled into the inter-prong grooves of the SLH domain structure. (*d*) Structure of the murein linkage unit that tethers the SCWP to the peptidoglycan of bacilli. (*e*) Model for the secretion and assembly of S-layer proteins in clostridia and bacilli, which appears to involve one or two major S-layer proteins as well as S-layer-associated proteins. Precursors can travel via the canonical secretory pathway involving SecA or a SecA2 pathway that appears dedicated for the secretion of S-layer proteins. The distribution of S-layer and S-layer-associated proteins is thought to be organized, albeit that the mechanisms for such organization have not yet been revealed.

Members of the *B. cereus* sensu lato group are rod-shaped, spore-forming bacteria [153]. The envelope of their vegetative forms is composed of a plasma membrane and peptidoglycan layer with attached SCWP [154] and capsules composed of polysaccharides, hyaluronic acid or poly-d-γ-glutamic acid [51,155–157]. The genome of *B. anthracis*, a member of the *B. cereus* sensu lato group, encompasses 24 open reading frames whose predicted translation products each contain a secretion signal and three tandem SLH domains [90,158] (figure 6). The *B. anthracis csaA*-*csaB*-*sap*-*eag* gene cluster encodes the two S-layer proteins, surface array protein (Sap) and extractable antigen 1 (EA1) [148,159,160], as well as CsaB, a pyruvyl transferase responsible for decorating SCWP with ketal-pyruvate [22,53]. *Bacillus anthracis* SCWP is a polymer with the repeating structure [→6)-α-GlcNAc-(1→4)-β-ManNAc-(1→4)-β-GlcNAc-(1→]*_n_*, where α-GlcNAc is substituted with α-Gal and β-Gal at O3 and O4, respectively, and the β-GlcNAc is substituted with α-Gal at O3 [52] (figure 6*b*). The SCWP is linked to the peptidoglycan layer via murein linkage units [53], i.e. a GlcNAc-ManNAc moiety that is phosphodiester linked to the C6 hydroxyl of MurNAc [16] (figure 6*d*). *Bacillus anthracis* forms S-layers from both Sap and EA1 by tethering the SLH domains of these polypeptides to pyruvylated SCWP [53,159,161] (figure 6*a*–*c*). C-terminal to the SLH domains, Sap and EA1 harbour crystallization domains that engage in recriprocal subunit–subunit interactions to constitute the S-layer [159,162,163] (figure 6*e*). A current model for S-layer assembly is that secreted subunits are recruited to the edge of an extant S-layer network via enthalpy-driven interactions between crystallization domains and are then tethered to the SCWP via the SLH domains [22]. This model matches growth of the S-layer(s) with increases in the avidity of these networks for the cell wall. This model can also explain how S-layers assemble on top of the peptidoglycan layer and thread capsule between individual S-layer subunits [51,160].

The overall structure of the three SLH domains resembles a three-pronged spindle, where each prong is derived from a single SLH domain [164] (figure 6*a*). The base of the spindle is assembled from all three domains, each of which contributes a single helix that associates into a three-helical bundle [164]. As each of the three SLH domains assumes a nearly identical fold, one can consider the entire structure as a pseudo-trimer. A group of five residues, designated the ITRAE motif for its consensus sequence, is partially conserved among the SLH domains of bacterial S-layer proteins [151]. The motif occupies the last four residues of loop B and the first residue of the central helix bundle. Within the SLH domains of *B. anthracis* Sap, these motifs have the sequences LTRAE, IDRVS and VTKAE, and contain the cationic residues Arg^72^, Arg^131^ and Lys^193^, respectively [164]. The corresponding positively charged residues of the ITRAE motif are necessary for the incorporation of SLH domain proteins into the S-layer [164].

A *B. anthracis csaB* mutant, which cannot pyruvylate the SCWP, is unable to assemble secreted Sap or EA1 into S-layers and forms elongated chains of vegetative forms that fail to separate [22]. This phenotype suggests a key function of S-layer and S-layer-associated proteins during the cell cycle of bacilli. The *csaB* phenotype suggests further that proteins within the S-layer may not be randomly distributed but rather assume discrete positions to fulfil their function. For example, the S-layer-associated protein BslO is deposited near the cell wall septa of bacilli [165]. Mutants lacking *bslO* display an elongated chain phenotype that can be complemented *in trans* with purified BslO. The glucosaminidase domain of BslO is thought to cleave septal peptidoglycan to promote the separation of vegetative forms [165]. The mechanism whereby BslO is localized to the septal portion of the bacillus S-layer has not yet been elucidated. *Clostridium difficile*, an anaerobic microbe that also forms spores, encodes two *secA* genes, one of which (*secA2*) is required for assembly of its S-layer proteins and the cell wall protein CwpV [166]. It is not yet clear how SecA2 selects S-layer protein precursors for secretion and why this mechanism contributes to S-layer assembly. Nevertheless, other S-layer producing microbes, for example, *B. anthracis*, also harbour *secA2* genes [158]. Thus, it is conceivable that S-layer assembly in many microbes involves a dedicated secretion pathway for the abundant transport of S-layer proteins with SLH domains.

Not all bacterial S-layers are assembled from proteins with SLH domains. The SbsC protein of *Geobacillus stearothermophilus* is an example for a class of protein that forms S-layers without SLH domains [167]. SbsC binds to the SCWP of *G. stearothermophilus* via its N-terminal domain, which consists of three triple-helical bundles connected by two contiguous helices [167]. The N-terminal domain of SbsC has high similarity with S-layer proteins from *G. stearothermophilus*, *Geobacillus kaustophilus* and *Geobacillus tepidamans*, suggesting that its mechanisms of assembly are conserved in other microbes [167].

## 11. Type VII secretion systems in Gram-positive bacteria

**Figure 7.**
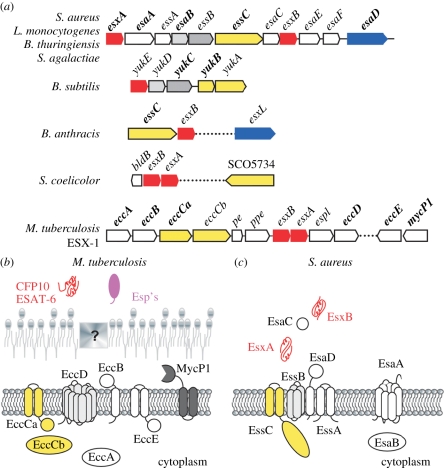
Genetic organization and models for WXG100 protein secretion in *M. tuberculosis* and *S. aureus.* (*a*) Gene clusters showing WXG100 products (in red) along with predicted FtsK-SpoIIIE ATPases (in yellow) are shown. The top diagram uses the nomenclature established experimentally for *S. aureus* and depicts *ess* genes required for ESAT-6-like secretion, *esa* genes playing an accessory role for ESAT-6 like secretion and *esx* genes encoding WXG100 proteins (ESAT-6 like). Gene names shown shaded represent putative plasma membrane proteins. Light and dark grey colours show genes conserved among Gram-positive bacteria while white colour is used when the function of the gene is unknown. The genetic organization for *M. tuberculosis* is shown only for ESX-1 (one of the five WXG100 secretion systems) and the nomenclature is as described by Bitter *et al*. [170]. Several Esp encoding genes encoded outside the ESX-1 cluster are not shown. (*b*,*c*) Models showing the cellular localization and predicted topology of conserved (Ecc), associated (Esp) and the MycP1 proteins of the ESX-1 gene cluster as well as conserved and accessory (Ess, Esa) proteins of the secretion system in *S. aureus*. The depiction of EccD and EssB as translocons is speculated based on prediction of transmembrane spanning sequences for these proteins. In *S. aureus,* the possible WXG100 translocon could be constituted of EssB, EsaD and EssA [171,172].

ESAT-6 (EsxA) and its homologue CFP-10 (EsxB) are small α-helical polypeptides and founding members of the WXG100 motif family [168,169]. ESAT-6 (Early Secreted Antigen 6 kDa) and CFP-10 (Culture Filtrate Protein 10 kDa) are secreted by *Mycobacterium tuberculosis*. Mark Pallen first suggested that genes clustering with *esxA* and *esxB* in the genome of *M. tuberculosis* may represent a novel secretion system (figure 7) [168]. This conjecture was proven to be correct when mutations in this gene cluster caused secretion defects for ESAT-6 and CFP-10 [173–175]. Nevertheless, the molecular mechanisms and the biochemical identity of the proposed ESAT-6 secretion machinery responsible for WXG100 protein secretion have not been revealed. Available models are derived from genetic variations and observations of mutant phenotypes when specific genes in the ESAT-6 and CFP-10 clusters are disrupted (figure 7*b*) [170]. It was recently suggested that ESAT-6 secretion should be referred to as a type VII secretion system [170,176]. The numerical classification is derived from Gram-negative bacteria, where polypeptides are transported across double membrane envelopes using mechanisms that are either independent of or expand the canonical Sec pathway [177]. Mycobacterial cell walls also encompass a double membrane envelope, including the plasma membrane and the mycolic acid layer with long aliphatic lipids [178–180]. Thus, the term type VII secretion appeared to fit with the previously discovered type I–VI pathways [170]. Nevertheless, as already noted by Pallen, genes encoding for putative WXG100 proteins are also found in the genomes of Gram-positive bacteria lacking double membrane envelopes (figure 7*a*) [168]. For example, two small WXG100 proteins, EsxA and EsxB, are secreted by *S. aureus* in a manner depending on genes that are clustered with *esxA* and *esxB* [171,172,181]. This gene cluster has been named for its function: ESAT-6 secretion system (Ess) [171] and a model for protein secretion were again derived from the genetic analysis of mutations in this cluster (figure 7*c*) [171,172]. It is difficult to draw parallels and commonalities between the WXG100 secretion systems for mycobacteria and staphylococci. Although WXG100 proteins carry a typical amino acid WXG signature, they share very little overall identity. Their striking similarity lies in the α-helical hairpin structure adopted by these proteins [169,182,183]. Further, only genes specifying for a predicted ATPase with FtsK-SpoIIIE domain are shared between staphylococcal Ess and mycobacterial T7SS. In mycobacteria, one of the ATPases appears to select substrates for secretion, whereas in *S. aureus* it is simply required for the secretion of EsxA and EsxB. Unlike CFP-10, where a C-terminal sequence was shown to be necessary for the secretion of CFP-10:ESAT-6 complexes [184], such an element has not be observed in staphylococcal EsxA and EsxB (M. Anderson 2011, personal communication). Other mycobacterial components, among them the EspACD proteins, also seem necessary for the secretion of ESAT-6 and CFP-10 [185]. Even more intriguing is the disparity in genetic composition of putative Ess pathways among mycobacteria and Gram-positive bacteria. Mycobacteria, but perhaps not other Gram-positive bacteria, harbour gene clusters that appear to encode five distinct type VII secretion systems (ESX1-5) [186]. Of these, the ESX-4 system may be the simplest system whose components also display the highest degree of conservation with the type VII systems of other Gram-positive bacteria [186]. Experimental proof for WXG100 protein secretion has thus far been garnered in two other Gram-positive organisms, *B. anthracis* and *S. coelicolor. B. anthracis* encodes six proteins with a WXG100 domain, only one of them, Ba-EsxB, is as short as ESAT-6 or CFP-10 (90 amino acids). This protein does not require the FtsK-SpoIIIE ATPase in the Ess cluster for its secretion [187]. However, Ba-EsxB is essential for the secretion of Ba-EsxW, a protein with an N-terminal WXG100 domain and a large C-terminal domain of unknown function encoded outside the *B. anthracis* Ess cluster. Intriguingly, only *B. anthracis* appears to encode WXG100 proteins with large C-terminal extensions. Several pathogenic bacilli (*B. cereus* and *B. thuringiensis*) encode an Ess-like gene cluster, whereas the non-pathogenic *B. subtilis* encodes a minimal Ess gene cluster with only one WXG100 gene (*yukE*), a split FtsK-SpoIIIE ATPase gene and *essB esaB* like genes (*yukC* yukD) (figure 7*a*). The *S. coelicolor* WXG100 proteins EsxA and EsxB have also been shown to be secreted [188]. S*treptomyces esxA* and *esxB* genes are located on a region of the chromosome that includes the regulator BlbD and the FtsK-SpoIIIE ATPase. Other genes within this cluster encode for proteins with domains of unknown function. *Streptomyces coelicolor* EsxA and EsxB are involved in the morphogenetic development that supports aerial hyphae and spore formation [188].

Loss of ESAT-6 and CFP-10 secretion affects the ability of *M. tuberculosis* to replicate in macrophages and to suppress innate and adaptive immune responses [175,189–192]. Loss of Ess-dependent secretion in *S. aureus* affects the developmental programme of abscess formation and staphylococcal persistence in host tissues [181]. A function for WXG100 proteins in *B. anthracis* could not be deduced. In spite of their dissimilarities, Ess or type VII secretion systems in mycobacteria and Gram-positive bacteria must share some functional properties when supporting the secretion of WXG100 proteins with similar structure. Nevertheless, Ess/type VII pathways certainly fulfil different functions, considering their wide distribution among Gram-positive bacteria and lack of a convergent phenotypic trait for mutants in this pathway in different bacteria. Much remains to be discovered regarding the molecular mechanisms that support substrate recognition, secretion or the pathophysiological attributes of the secreted products. Considering that conserved genes (FtsK/SpoIIIE type ATPase and WXG-100 proteins) are shared among bacterial pathogens that generate distinctive disease features, the effectors of type VII and type VII-like secretion systems may be proteins that do not belong to the WXG-100 family, as has been reported for *M. tuberculosis* and *S. aureus* [172,193].
